# Supplementary material for: Effects of the non-parabolic kinetic energy on non-equilibrium polariton condensates
Source: Sci Rep. 2017 May 15;7:1891. doi: 10.1038/s41598-017-01113-8 (PMC5432531; doi:10.1038/s41598-017-01113-8)
Supplement: Supplementary file 1 — Supplemental material [file 41598_2017_1113_MOESM1_ESM.pdf]

Supplemental material: Effects of the non-parabolic kinetic energy on non-equilibrium polariton condensates

F. Pinsker,[1] X. Ruan,[2] and T. J. Alexander[3]

[1] *Clarendon Laboratory, Department of Physics, University of Oxford, Parks Road, Oxford OX1 3PU, United Kingdom.*[*]

[2] *Department of Mathematics, National University of Singapore, Singapore.*

[3] *School of Physical, Environmental and Mathematical Sciences, UNSW Canberra, Canberra ACT 2600, Australia.*

(Dated: February 16, 2017)

Plane waves

In this section we discuss some of the simpler plane wave results and predictions which underpin the full analysis presented in the Results section, and present a number of other related plane wave results. We begin with the simplest case first and so assume the condensate to be unpolarised and so well described by a single mean-field equation. Later we will add different aspects relevant for a more accurate understanding of a condensed polariton wave.

To derive the plane wave solutions and to gain intuition about the mathematical structure of the polariton kinetic energy we first take the Fourier transform of the simplest time dependent differential equation

$$i\partial_t\psi = q\star\psi \equiv \mathcal{F}^{-1}(\omega_{\mathrm{L,U}}(\mathbf{k})\mathcal{F}(\psi)) \tag{1}$$

i.e.,

$$i\partial_t\mathcal{F}(\psi) = \omega_{\mathrm{L,U}}(\mathbf{k})\mathcal{F}(\psi). \tag{2}$$

It has an explicit solution

$$\mathcal{F}(\psi) = \exp(-i\omega_{\mathrm{L,U}}(\mathbf{k})t). \tag{3}$$

On the other hand we note that for

$$i\partial_t\psi = -\gamma\Delta\psi \equiv \gamma\mathcal{F}^{-1}(|\mathbf{k}|^2\mathcal{F}(\psi)) \tag{4}$$

we obtain the solution

$$\mathcal{F}(\psi) = \exp(-i\gamma|\mathbf{k}|^2 t). \tag{5}$$

Comparing (3) and (5) we observe that the stronger the dispersion deviates from the approximation given in (5) by $\gamma|\mathbf{k}|^2$ the more the phase evolves at a different rate. Note that a constant offset is achieved by

$$\mathcal{F}(\psi) = \exp(-i(\gamma|\mathbf{k}|^2 + \omega_0)t), \tag{6}$$

which solves the more general PDE

$$i\partial_t\psi = (-\gamma\Delta + \omega_0)\psi. \tag{7}$$

Note that Eq. 7 corresponds to a mean-field polariton equation in the parabolic approximation without interactions and non-equilibrium processes. Note also that the generalised derivative is defined by

$$(-\Delta)^s f(\mathbf{r}) \equiv \mathcal{F}^{-1}(|\mathbf{k}|^{2s}\mathcal{F}(f)) = \frac{1}{(2\pi)^d}\int_{\mathbb{R}^d} |\mathbf{k}|^{2s}\hat{f}(\mathbf{k})e^{i\mathbf{r}\cdot\mathbf{k}}\mathrm{d}\mathbf{k} \tag{8}$$

For $s = 1$ one considers the cGPE with parabolic dispersion. It has been noted in [**?**] that a fractional kinetic energy is a closer approximation to the polariton dispersion, in the Euclidean sense, than the parabolic approximation.

Now by using properties of the Fourier transform, we have

$$(q\star e^{i\mathbf{k}_{\mathrm{i}}\cdot\mathbf{r}})e^{-i\mathbf{k}_{\mathrm{i}}\cdot\mathbf{r}} = \mathcal{F}^{-1}\left(\hat{q}(\mathbf{k})(2\pi)^d\delta(\mathbf{k}-\mathbf{k}_{\mathrm{i}})\right)e^{-i\mathbf{k}_{\mathrm{i}}\cdot\mathbf{r}} = \hat{q}(\mathbf{k}_{\mathrm{i}}), \tag{9}$$

which implies our ansatz for the spatial variation of the wave function in the following analysis. Let us consider the scenario of a stationary mean-field polariton state with self-interactions stemming from the polariton-polariton scattering and constant interactions with the reservoir n_R, i.e. we consider the PDE

$$q\star\psi + \alpha_1|\psi|^2\psi = (\mu - \alpha_1 n_R)\psi. \tag{10}$$

The ansatz for a plane wave solution is $\psi(\mathbf{r},t) = \phi_0\exp(i\mathbf{k}_{\mathrm{i}}\cdot\mathbf{r})$ and by recognising the translation property of the Fourier transform $\mathcal{F}(\psi) = \phi_0\delta(\mathbf{k}-\mathbf{k}_{\mathrm{i}})$ we write (10) as

$$\left[\omega(\mathbf{k}_{\mathrm{i}}) + \alpha_1(|\phi_0|^2 + n_R)\right] = \mu. \tag{11}$$

It has the analytical solutions

$$\phi_0 = \pm\sqrt{\frac{\mu - \alpha_1 n_R - \omega(\mathbf{k}_\mathrm{i})}{\alpha_1}} \tag{12}$$

up to a constant phase factor and thus the plane wave rays for the explicitly stationary case is given by

$$\psi(\mathbf{r}, t) = \pm\sqrt{\frac{\mu - \alpha_1 n_R - \omega(\mathbf{k}_\mathrm{i})}{\alpha_1}} \exp(i\mathbf{k}_\mathrm{i} \cdot \mathbf{r}) \exp(-i\mu t/\hbar). \tag{13}$$

Note that we have derived a corresponding solution for the full model in the main text, which in particular includes the pumping dynamics inherent in the description of the polariton condensate.

Adaption of the plane wave due to time dynamics

Consider the generalised dispersive PDE in 1+2d that describes the evolution equation for the polariton condensate with negligible interactions to the phonon bath and without pumping,

$$q \star \psi + \alpha_1 |\psi|^2 \psi = i\hbar \partial_t \psi. \tag{14}$$

We set $\psi = v(t) \exp(i\mathbf{k}_\mathrm{i} \cdot \mathbf{r})$ such that

$$q \star \psi = q \star (v(t) \exp(i\mathbf{k}_\mathrm{i}\mathbf{r})) = v(t) \cdot \mathcal{F}^{-1}(\omega(\mathbf{k})(2\pi)\delta(\mathbf{k} - \mathbf{k}_\mathrm{i})) = \omega(\mathbf{k}_\mathrm{i}) v(t) \exp(i\mathbf{k}_\mathrm{i} \cdot \mathbf{r}). \tag{15}$$

Hence ψ is an eigenfunction of the operator $q\star$, with eigenvalue $\omega(\mathbf{k}_\mathrm{i})$. On the other hand the dynamical part has to satisfy

$$i\hbar \partial_t v = (\omega(\mathbf{k}_\mathrm{i}) + \alpha_1 |v|^2) v, \tag{16}$$

which is solved by

$$v(t) = A \exp(-i\omega(\mathbf{k}_\mathrm{i}) t/\hbar) \exp(-i\alpha_1 |A|^2 t/\hbar). \tag{17}$$

So we obtain an explicitly time dependent plane wave solution

$$\psi(\mathbf{r}, t) = A \exp(-i\omega(\mathbf{k}_\mathrm{i}) t/\hbar) \exp(-i\alpha_1 |A|^2 t/\hbar) \exp(i\mathbf{k}_\mathrm{i} \cdot \mathbf{r}). \tag{18}$$

We point out that the time oscillation of $\exp(-i\omega(\mathbf{k}_\mathrm{i})t/\hbar)$ arising from the linear evolution is augmented by the time oscillation due to the nonlinear self-interactions, i.e. $\exp(-i\alpha_1 |A|^2 t/\hbar)$. Furthermore in [3] it has been pointed out (for simpler positive parabolic dispersions) that for the defocusing case $\alpha_1 > 0$, both time oscillations are clockwise, so one can view the defocusing nonlinearity as amplifying the dispersive effect of the linear equation. Furthermore note that in the focusing case the focusing nonlinearity is instead trying to cancel the dispersive effect. If the amplitude A is small compared the frequency ω then the dispersive effect is stronger, but when the amplitude is large then the focusing effect takes over [3].

Moving plane wave

Let us consider next the generalised dispersive PDE for a wave in motion with velocity $\mathbf{v}$, i.e.

$$q \star \psi + i\mathbf{v}\nabla\psi + \alpha_1 |\psi|^2 \psi = i\hbar \partial_t \psi. \tag{19}$$

First let us again make the separation of variables ansatz

$$\psi = p(t) \exp(i\mathbf{k}_\mathrm{i} \cdot \mathbf{r}), \tag{20}$$

which implies

$$i\mathbf{v}\nabla\psi = -\mathbf{k}_\mathrm{i} \cdot \mathbf{v}\psi. \tag{21}$$

On the other hand we have to satisfy

$$i\hbar\partial_t p = (\omega(\mathbf{k}_\mathrm{i}) + \alpha_1|p|^2 - \mathbf{k}_\mathrm{i}\cdot\mathbf{v})p. \tag{22}$$

This is solved by

$$p(\hbar t) = A\exp(-i\omega(\mathbf{k}_\mathrm{i})t)\exp(-i\alpha_1|A|^2 t)\exp(i\mathbf{k}_\mathrm{i}\cdot\mathbf{v}t) \tag{23}$$

and thus we obtain the explicit solution

$$\psi(\mathbf{r},t) = A\exp(-i\omega(\mathbf{k}_\mathrm{i})t/\hbar)\exp(-i\alpha_1|A|^2 t/\hbar)\exp(i\mathbf{k}_\mathrm{i}\cdot\mathbf{v}t/\hbar)\exp(i\mathbf{k}_\mathrm{i}\cdot\mathbf{r}). \tag{24}$$

We observe that once the plane wave is put into motion relative to the intertial reference frame the effective rotation of the phase can be canceled out or reversed to anti-clockwise. However note that the Galilean boosted wave function would include an addition factor such that the equation of motion has the same form in all inertial reference frames.

Excitation spectrum

The linearised elementary excitation equation around the stationary states without trapping and under incoherent pumping is stated in [1, 2] and the eigenvalues of the Bogoliubov operators for the excitation modes including the $\hat{q}(k)$ dependence are the generalised Bologliubov dispersions for linear excitations, i.e.

$$\omega_{\mathrm{Bog}}^{\mathrm{m(k)}}(k) = -i\frac{\Gamma}{2} \pm \left[\hat{q}(k)\left(\hat{q}(k) + \omega_\mathrm{i} + 2\alpha_1 n\right) - \frac{\Gamma^2}{4}\right]^{1/2} \tag{25}$$

with an effective damping rate

$$\Gamma = -(P(\mathbf{r},t) - \Gamma n - \gamma). \tag{26}$$

The signs correspond in (25) to the positive and negative Bogoliubov branch and as expected the presence of the velocity dependent mass adapts the excitation energy by a non-parabolic k dependence of the effective kinetic energy.

Free particle propagator

Note that the free particle propagator in momentum space corresponding to a generalized kinetic energy is given by

$$K_L^{(0)}(x_b t_b|x_a t_a) = \int \frac{dp}{2\pi}\cdot\exp\left\{i\frac{p(x_b - x_a)}{\hbar} - i\frac{(t_b - t_a)g(p/\hbar)}{\hbar}\right\}, \tag{27}$$

which is a natural extension of the free fractional quantum mechanics propagator [4] and includes this case for the specific choice $g(p) = D^\alpha|p|^\alpha$.

---

* florian.pinsker@physics.ox.ac.uk

[1] Carusotto, I. & Ciuti, C., Quantum fluids of light, Rev. Mod. Phys. **85**, 299 (2013).
[2] Wouters, M. & Carusotto, I., Excitations in a Nonequilibrium Bose-Einstein Condensate of Exciton Polaritons, Phys. Rev. Lett. **99**, 140402 (2007).
[3] Tao T., Nonlinear dispersive equations: local and global analysis, CBMS regional conference series in mathematics, (2006).
[4] Laskin, N., Fractional quantum mechanics and Levy paths integrals, Phys. Lett. A **268**, 4 – 6, pp. 298-305 (2000).
